# An Interpretable Biomimetic Auditory Framework for Target Speech Enhancement via Pitch-Synchronous Delay Superposition

**DOI:** 10.3390/biomimetics11100707

**Published:** 2026-10-04

**Authors:** Zhilei Liu, Lei Guan, Dayong Ning, Ziwei Li, Xiuming Yao, Jiaoyi Hou

**Affiliations:** Naval Architecture and Ocean Engineering College, Dalian Maritime University, Dalian 116026, China; liuzl@dlmu.edu.cn (Z.L.);

**Keywords:** target speech enhancement, delay superposition mechanism, selective attention, speech separation

## Abstract

The human auditory system can selectively prioritize a target speaker in the presence of competing speech, providing a biological model for the design of interpretable auditory systems. In this study, we present a biomimetic computational auditory framework that translates temporal-processing principles of human hearing into an engineering mechanism for target-speech enhancement. Motivated by the distinct temporal characteristics of transient and quasi-periodic speech components, the proposed framework employs two complementary processing pathways. A first-order differential pathway emphasizes transient consonant cues, whereas a delay-superposition pathway exploits the quasi-periodicity of vowel signals to reinforce target-related periodic structures. The enhanced components are subsequently screened through an enhancement-based criterion and selectively reconstructed using zero-crossing-based signal replacement. Experiments on CRM two-talker speech mixtures demonstrated effective target-speech enhancement across multiple speaker-pair conditions, including both same- and cross-gender mixtures. The proposed method consistently improved target selectivity and preserved the principal spectral characteristics of the designated speaker. Psychoacoustic experiments were further used to examine the perceptual relevance of differentiated consonant-vowel processing and selective auditory attention. Rather than reproducing auditory neural circuitry directly, the proposed model provides an interpretable engineering abstraction of biological temporal-selection principles. These findings demonstrate how auditory mechanisms can be translated into a transparent biomimetic signal-processing architecture and provide a basis for future bio-inspired and neuromorphic auditory systems.

## 1. Introduction

The human auditory system can effortlessly focus on a single speaker in crowded, noisy environments—a classic phenomenon known as the cocktail party problem [1,2,3]. Yet, despite decades of extensive investigation, the neural mechanisms underpinning such precise and robust auditory selection remain elusive [4,5,6]. This perceptual efficacy masks a set of intricate computational challenges: the brain must disentangle overlapping auditory streams and suppress diverse competing stimuli, ranging from background chatter to ambient acoustic clutter.

Consequently, utilizing biologically inspired models to interpret the cocktail party effect through neural simulation remains a pivotal research direction. Although traditional algorithms—such as spectral subtraction [7,8], Non-negative Matrix Factorization [9,10,11], and Independent Component Analysis (ICA) [12,13,14,15]—have achieved partial success in multi-source separation, they exhibit significant limitations in complex acoustic environments and dynamic noise scenarios. Driven by their strong ability to model complex nonlinear features, deep learning has emerged as the mainstream technology. For instance, Deep Clustering [16] characterizes time-frequency units for source grouping; Permutation Invariant Training (PIT) [17] resolves label ambiguity by minimizing mean squared error during backpropagation; Conv-TasNet [18] establishes an end-to-end framework for time-domain speech separation. It employs a linear encoder to generate representations of the speech waveform, which are optimized to enable the effective separation of individual speakers. However, while defining the state-of-the-art, deep neural networks are constrained by intrinsic limitations, particularly the lack of interpretability in their latent representations [19]. Models often function as opaque ‘black boxes’ that prioritize statistical mapping over the integration of acoustic physics or biological auditory principles. Consequently, this neglect of explicit physiological mechanisms limits our insight into the model’s decision-making process, thereby restricting the potential to improve robustness and generalization through biologically plausible constraints.

To address the limitations of neural network models, researchers have sought to optimize performance by drawing inspiration from the human brain’s auditory processing mechanisms. By integrating interpretable bio-inspired signal processing methods with deep learning frameworks, these approaches mimic peripheral auditory processes—such as cochlear frequency decomposition and non-linear compression—alongside the extraction of cortical spectro-temporal modulation features [20]. Experiments demonstrate that such models can be trained effectively with limited data and exhibit superior robustness in noisy environments compared to purely data-driven models. Furthermore, by delving into cochlear perceptual characteristics, researchers have employed the CAR-FAC cochlear model to simulate auditory function for sound localization in reverberant environments [21]. By incorporating the Haas effect, these systems effectively suppress echo interference, ensuring accurate localization within complex acoustic scenes. This line of research not only preserves the physiological interpretability of bio-inspired methods but also leverages neural networks to optimize performance for specific task requirements. However, existing bio-inspired models primarily focus on peripheral filtering or spatial localization, often overlooking the central nervous system’s hierarchical processing strategy—specifically, the differential neural encoding of transient (consonant) versus steady-state (vowel) components. Furthermore, although the Haas effect has primarily been studied in spatial hearing and de-reverberation, its emphasis on the perceptual importance of relative timing between leading and delayed acoustic components provides useful inspiration for exploring delay-based processing strategies in speech enhancement.

To construct such a biologically grounded architecture, we must first look to the organizational principles of the human auditory system. Neuroscientific research indicates that selective listening involves hierarchical processing along the auditory neural axis, from the primary segregation mediated by the brainstem to the semantic analysis in the cerebral cortex. At the cortical level, selective listening to competing speech has been associated with attentional modulation and entrainment of cortical oscillations [22]; The precedence (Haas) effect [23,24,25] further demonstrates that auditory perception is strongly influenced by the relative timing between leading and delayed acoustic components. Within short inter-sound delays, the delayed component can contribute to a fused percept dominated by the leading sound, illustrating the importance of temporal relationships in auditory scene organization. Continuous speech is also represented in the auditory cortex through phase-locked temporal coding of slow speech modulations [26]. Electrocorticographic recordings have further shown that non-primary auditory cortex selectively represents salient spectro-temporal features of an attended speaker under multi-talker conditions [27]. This hierarchical processing model partially explains the neural basis of the “cocktail party effect” [28]. However, the implementation mechanism of selective attention has not been thoroughly elucidated: Does the neural dissociation mechanism of vowels and consonants during selective listening show differences? Do all sound components in noisy auditory signals undergo a complete neural dissociation process? How can the nervous system realize the analysis of multi-source acoustic scenes and complete the reconstruction of the sound of concern?

Based on these observations, we develop a biomimetic computational auditory framework that translates selected temporal-processing principles of human hearing into an interpretable engineering mechanism for target-speech enhancement. The framework incorporates two complementary processing pathways for transient consonantal features and quasi-periodic voiced components. The enhanced components are subsequently screened and recombined to reconstruct the target speech.

From a signal-processing perspective, the proposed DSA is related to established pitch-dependent processing methods. Pitch-adaptive speech-enhancement methods exploit pitch-related temporal correlation and harmonic structure, while comb-filter-based speech-separation methods use periodicity-dependent filtering to enhance or separate speech components [29,30]. Modern time-domain source-separation systems, such as Conv-TasNet [18], instead learn end-to-end latent representations for separating competing speakers. The methodological contribution of the present framework lies in integrating pitch-related delay superposition with a complementary transient-processing pathway, local multi-delay candidate searching, enhancement-based segment screening, and selective waveform reconstruction. Thus, delay superposition is used as one component of an interpretable dual-pathway target-speech enhancement framework rather than as a standalone harmonic filter.

From a biomimetics perspective, the purpose of the proposed framework is not to reproduce the complete neural circuitry underlying selective hearing, but to abstract biologically observed auditory-processing principles into a transparent and quantitatively testable computational architecture. In this way, the biological inspiration is explicitly translated into engineering operations that can be evaluated in a controlled speech-separation task.

The main contributions of this study are summarized as follows:

(1) A dual-pathway biomimetic auditory-processing framework is developed to treat transient consonant features and quasi-periodic vowel components using complementary computational mechanisms, providing an interpretable alternative to purely data-driven speech-separation approaches.
(2) A time-domain delay-superposition algorithm inspired by the delay-dependent perceptual characteristics associated with the Haas effect is introduced to selectively reinforce periodic target-speech components while suppressing mismatched interference.
(3) Signal-based evaluation and psychoacoustic experiments are combined to examine the functional correspondence between the proposed computational strategy and selected characteristics of human auditory perception, thereby demonstrating how biological auditory principles can inform the design of interpretable speech-processing systems.

## 2. Methods

### 2.1. Theoretical Foundation and Computational Framework

The resolution of complex acoustic scenes by the human auditory system relies on the precise encoding and integration of spectro-temporal features. Existing neurophysiological evidence indicates that the processing of speech signals in auditory centers is not a unidimensional linear process; rather, it involves differential processing based on the heterogeneity of acoustic features. Specifically, the auditory brainstem and early cortical networks exhibit distinct neural response patterns for transient components (e.g., consonants) and periodic steady-state components (e.g., vowels) within speech. This mechanism enables the auditory system to capture rapidly changing acoustic edges within extremely short time windows, while simultaneously leveraging temporal integration mechanisms to enhance the representation of periodic signals.

**Figure 1:**
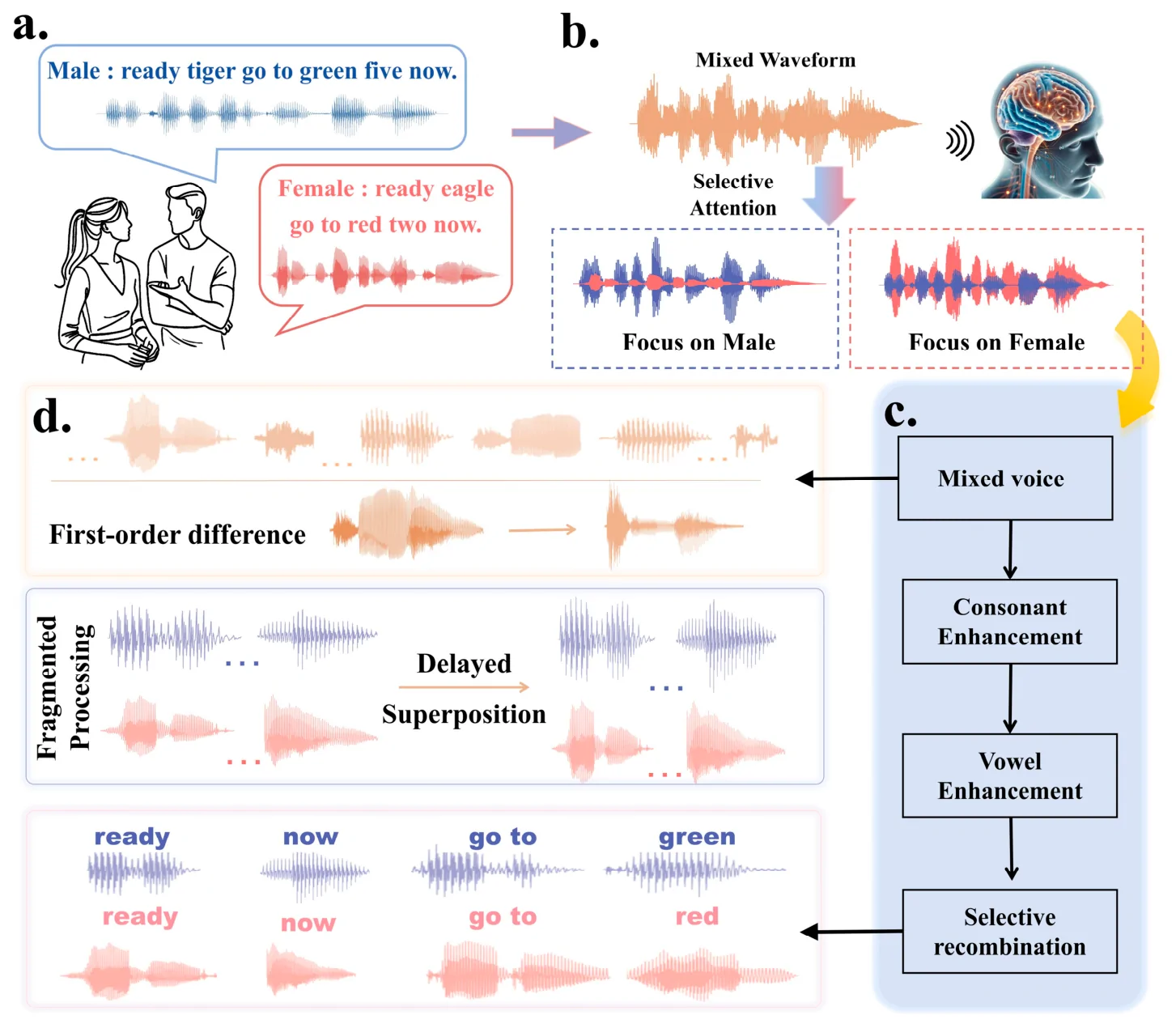
**Schematic of the proposed bio-inspired signal-processing framework for target-speech enhancement.** (**a**) Multi-speaker mixed speech input. (**b**) Target-selection objective: the framework enhances the acoustic representation of one designated speaker while suppressing competing speech. (**c**) Processing architecture consisting of transient-feature enhancement, periodicity-based enhancement, and selective reconstruction. (**d**) Signal-domain implementation using first-order differencing for rapidly varying components and pitch-synchronous delay superposition for quasi-periodic voiced components. The illustrated operations represent engineering abstractions and are not intended as direct models of neural waveform processing.

Figure 1 summarizes the proposed bio-inspired signal-processing framework. The mixed speech signal is processed through two complementary pathways that handle transient speech features and periodic components, respectively, followed by enhancement-based segment screening and selective reconstruction. The framework is motivated by selected temporal characteristics of speech and auditory perception rather than by a direct model of neural computation. As shown in Figure 1, the proposed framework consists of three main processing stages. First, transient consonantal features are enhanced through the consonant-processing pathway. Second, the quasi-periodic characteristics of voiced components are exploited by the delay-superposition pathway. Finally, candidate enhanced segments are screened and selectively reconstructed to obtain the target-speech output. The detailed implementation of these processing stages is presented in Section 3.2.1, Section 3.2.2 and Section 3.2.3.

Importantly, the signal representation used in the present framework is an engineering representation rather than a physiological one. The proposed algorithm operates directly on sampled acoustic waveforms for computational convenience. In biological hearing, however, acoustic signals undergo frequency-selective cochlear processing, hair-cell transduction, and auditory-nerve encoding before being represented in central auditory structures [31]. Therefore, the first-order-difference and delay-superposition operations proposed here constitute transparent signal-processing abstractions motivated by selected temporal characteristics of speech and auditory perception, rather than literal models of cochlear, brainstem, or cortical computation.

### 2.2. Delay Superposition Algorithm

To effectively extract vowel components from mixed speech signals, this study leverages the significant Fundamental Pitch Period and Quasi-periodicity characteristics of speech to propose a robust Delayed Superposition Algorithm (DSA). This algorithm aims to enhance target vowel features while suppressing non-periodic interference through coherent superposition in the time domain. Its specific implementation comprises three core stages: delayed superposition, extreme value optimization, and intelligent splicing.

#### 2.2.1. Signal Model and Periodicity Assumption

Assume that the observed discrete mixed signal $A_{mix}[n]$ is the linear superposition of the target speaker signal $A_{t}[n]$ and the interference signal $A_{i}[n]$. Its mathematical model is expressed as:

(1) $$A_{mix}[n]\;={\;A}_{t}[n]\;+\;A_{i}[n]$$

where n denotes the discrete time index (sample point index). Based on the physical mechanism of vocal fold vibration, vowel signals exhibit a high degree of periodicity under the short-time stationarity assumption. If the fundamental pitch period of the target speech is *T* (unit: number of samples), the target signal satisfies the following approximate periodicity characteristic:

(2) $$A_{t}[n]\;≅\;A_{t}[n\;+\;mT],\;m\in Z$$

This approximation indicates that the vowel waveform repeats after integer multiples of the fundamental pitch period, providing a solid theoretical basis for utilizing delayed coherent superposition to enhance the target component. Here, *T* should be interpreted as a local pitch-period parameter rather than as a constant value for the entire utterance. Because the fundamental frequency of natural speech varies with time, the quasi-periodicity assumption is applied only over locally voiced segments under the short-time stationarity approximation.

#### 2.2.2. Iterative Delayed Superposition Mechanism

Based on the aforementioned characteristics, the proposed algorithm employs an iterative shifting approach to cumulatively superpose the mixed signal. The enhanced signal $A_{enh}^{\left(k\right)}[n]$ after the *k*-th superposition is defined as:

(3) $$A_{enh}^{\left(k\right)}[n]\;=\;\sum_{j=0}^{k}A_{mix}[n\;+\;jT],\;k\;=\;0,\;1,\;2,\ldots ,\;K\;$$

To reduce computational complexity, this process can be expressed in a recursive form:

(4) $$A_{enh}^{\left(k\right)}[n]\;=\;A_{enh}^{(k-1)}[n]\;+{\;A}_{mix}[n+kT]$$

where *K* represents the preset maximum number of superposition iterations, typically ranging from 2 to 5 to strike a balance between enhancement performance and computational overhead. It should be noted that the present formulation is intended for offline batch processing. Because Equation (3) accesses future samples at n + jT, the current implementation is non-causal and requires a look-ahead of up to *KT* samples. Under the parameter settings used in this study (*K* ≤ 5, *T* ≤ 500 samples, and a sampling rate of 40 kHz), the maximum algorithmic look-ahead is 2500 samples, corresponding to approximately 62.5 ms. A causal or low-latency implementation could be developed using buffered processing or past-period superposition, but such a streaming implementation was not evaluated in the present study. Since the delay length *T* corresponds to the fundamental period of the target speech, the target speech component $A_{t}[n]$ remains highly coherent with itself after delays that are integer multiples of *T*, resulting in constructive interference and a linear increase in amplitude. In contrast, the interference speech component $A_{i}[n]$, whose period does not match *T*, undergoes destructive interference during the delayed superposition process, leading to significant energy suppression. Thereby, this procedure adaptively extracts and enhances the target speaker’s component from the mixed signal.

#### 2.2.3. Extreme Value Screening Optimization

To avoid errors introduced by blind superposition and to screen out speech segments with significant enhancement effects, the algorithm introduces a decision criterion based on local amplitude comparison. Define *E*(*n*) as the set of amplitude extrema of the enhanced signal in the vicinity of position n, and *M*(*n*) as the corresponding extrema set of the original mixed signal. A segment is judged as a valid enhanced segment and retained if and only if the following condition is met:

(5) $$\underset{p\in E(n)}{\max}|A_{enh}[p]|\;\geq \;N\cdot \underset{p\in M(n)}{\max}|A_{mix}[p]|$$

where *p* denotes the index of the extreme point, and N is the amplitude gain threshold (in this study, we set *N* = 2~3). This step ensures that reconstruction is only performed in regions where the target signal energy achieves a significant gain, effectively eliminating segments with insufficient signal-to-noise ratio (SNR) improvement.

#### 2.2.4. Waveform Reconstruction Based on Zero-Crossing Points

In the signal reconstruction phase, direct time-domain splicing is prone to inducing high-frequency transient noise. To reduce abrupt waveform discontinuities, the proposed algorithm restricts signal replacement to negative-going zero-crossing points (ZCPs). The selected zero-crossing point *n_c_* must simultaneously satisfy the following two conditions:

(6) $$A_{mix}[n_{c}]\cdot A_{mix}[n_{c}+1]\;\leq \;0$$

(7) $$A_{mix}[n_{c}]\;<\;0$$

Equation (6) identifies waveform zero crossings, and Equation (7) further restricts the selected boundary to a negative-going zero crossing. In the present implementation, these points are used as low-amplitude replacement boundaries to reduce abrupt amplitude discontinuities during segment reconstruction. However, zero-crossing alignment alone does not guarantee strict phase continuity between the original and enhanced waveforms. Therefore, the present strategy should be regarded as a simple boundary-selection heuristic rather than a phase-matched splicing procedure.

For each retained candidate segment, the corresponding replacement interval is bounded by the selected zero-crossing points in the original mixed waveform. Samples outside this interval remain unchanged, whereas samples within the interval are replaced by the corresponding delay-superposition-enhanced segment. No additional cross-fading or overlap-add operation is applied in the present implementation. Therefore, residual click-like artifacts or local phase discontinuities may still occur, particularly for non-stationary speech. More advanced boundary-smoothing strategies, such as short-window Hann/Hamming cross-fading or overlap-add reconstruction, will be considered in future work.

For clarity and reproducibility, the complete processing procedure of the proposed DSA is summarized in Algorithm 1.


**Algorithm 1. Processing procedure of the proposed DSA**
**Input:** mixed speech x[n], candidate delay range $\left[T_{\min},T_{\max}\right],$ enhancement threshold *N*, maximum superposition count *K***Output:** reconstructed target-enhanced signal *y*[*n*]1Initialize y[n] ← x[n].
2Search candidate delays *T* within $\left[T_{\min},T_{\max}\right].$
3For each candidate TT, perform iterative delay superposition according to Equations (3) and (4), for k = 1,…,K.
4Detect corresponding local extrema in the enhanced and original mixed signals.5Retain candidate segments satisfying the amplitude-enhancement threshold *N*.6Determine the corresponding zero-crossing boundaries for each retained segment.7Replace the samples of *y*[*n*] within the selected interval by the corresponding enhanced candidate segment; preserve the original mixture outside the interval.8Repeat for all retained segments and return *y*[*n*].

## 3. Experimental Setup and Performance Evaluation

Having established the theoretical framework of the DSA in the previous chapter, this chapter focuses on its experimental validation and performance assessment. We begin by detailing the data preparation and preprocessing protocols using standard speech corpora. Subsequently, we describe the specific algorithmic implementation of the hierarchical processing architecture and the complete experimental workflow. Finally, we present a comprehensive quantitative analysis of the separation results, evaluating the model’s effectiveness in enhancing target speech intelligibility against multi-speaker interference.

### 3.1. Experimental Data Processing

Utilize voice samples from the public database Coordinate Response Measure (CRM), which contains sentences in the form of “ready (call sign) go to (color) (number) now”. Select one male and one female speaker (Speakers 1 and 5 in the CRM corpus). Specifically, select the male speaker with the call sign “tiger”, the color “green” and the number “five”, and the female speaker with the call sign “eagle”, the color “red” and the number “two” for mixing. The selection of mixed samples ensures that there is no overlap in the call signs, colors, or numbers between the two phrases.

In addition to the primary two-speaker evaluation described above, we conducted an additional multi-speaker evaluation to examine the robustness of the delay-superposition strategy across different speaker pairs. Six speaker pairs were selected to cover male-male, female-female, and male-female combinations, and three different speech mixtures were generated for each pair. Each speaker was alternately designated as the target, resulting in 36 target-specific evaluation trials. Because the proposed method does not require model training, a conventional training partition was not required. For this additional evaluation, one independent enrollment utterance from each designated target speaker was used to estimate the speaker-specific pitch-period cue *T*, while separate utterances were reserved for evaluation, thereby preventing overlap between the delay-estimation and evaluation utterances.

### 3.2. Implementation of Hierarchical Processing

#### 3.2.1. Consonant Processing Method

**Figure 2:**
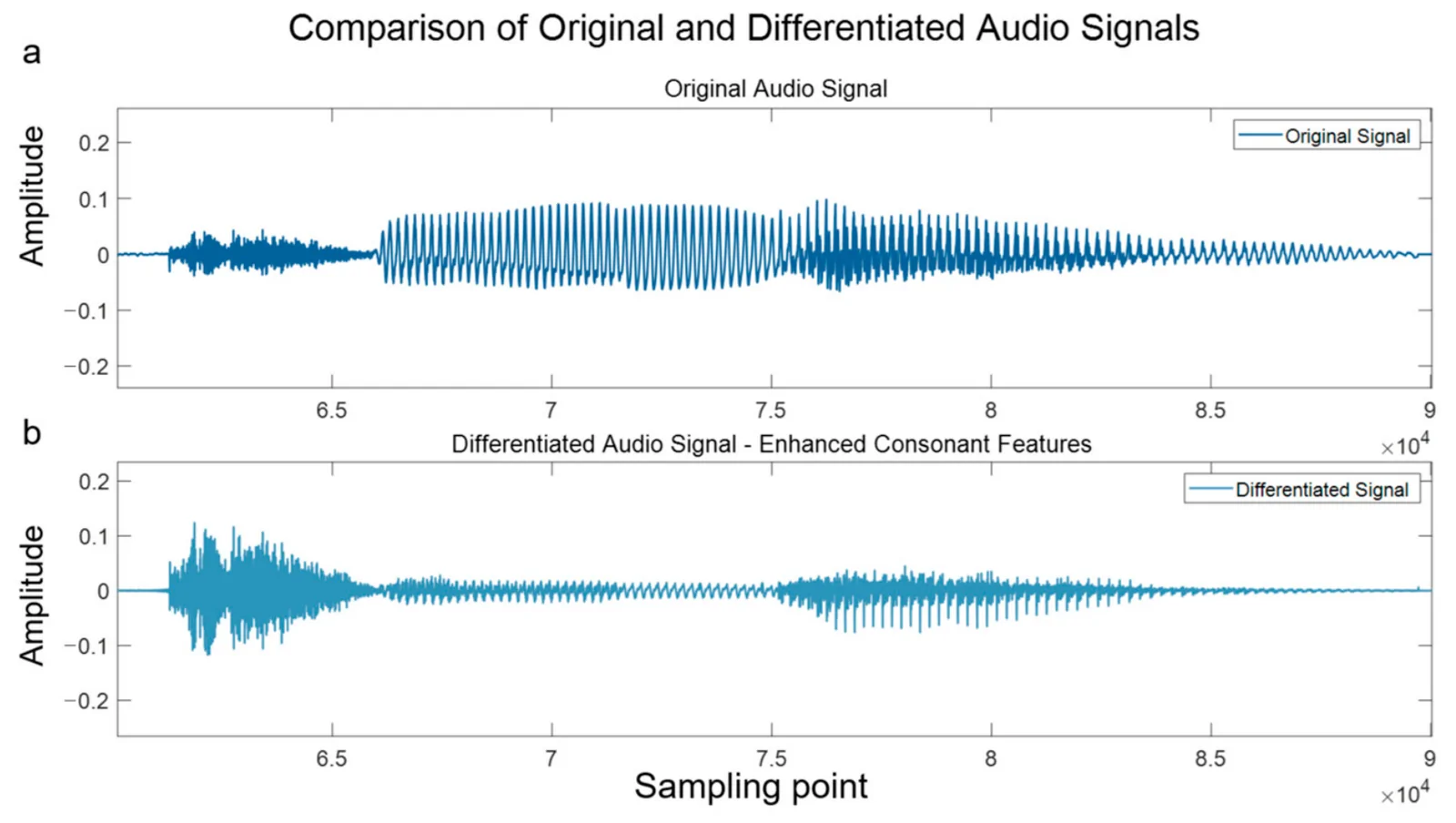
**Changes in the characteristics of mixed audio signals before and after differential processing.** The original signal (**a**), where the energy difference between consonants and vowels is evident; The signal after differential processing (**b**). In this waveform, the characteristics of consonants (such as instantaneous amplitude) change more significantly, manifesting as more pronounced spikes.

Given that consonants are characterized by low energy and high-frequency transient components, we employed a first-order differential method to enhance their observability. This process acts as a pre-emphasis filter, flattening the spectral tilt and boosting high-frequency energy. The operation is defined as:

(8) d[n] = α·(x[n]−x[n−1])

where x[n] represents the discrete mixed signal, x[n−1] is the unit-delayed signal, and α is a gain scaling factor used to compensate for the amplitude reduction caused by differencing. As illustrated in Figure 2, this operation effectively highlights the transient edges of consonants, making them distinguishable from the high-energy vowel background.

#### 3.2.2. Vowel Processing Method

**Figure 3:**
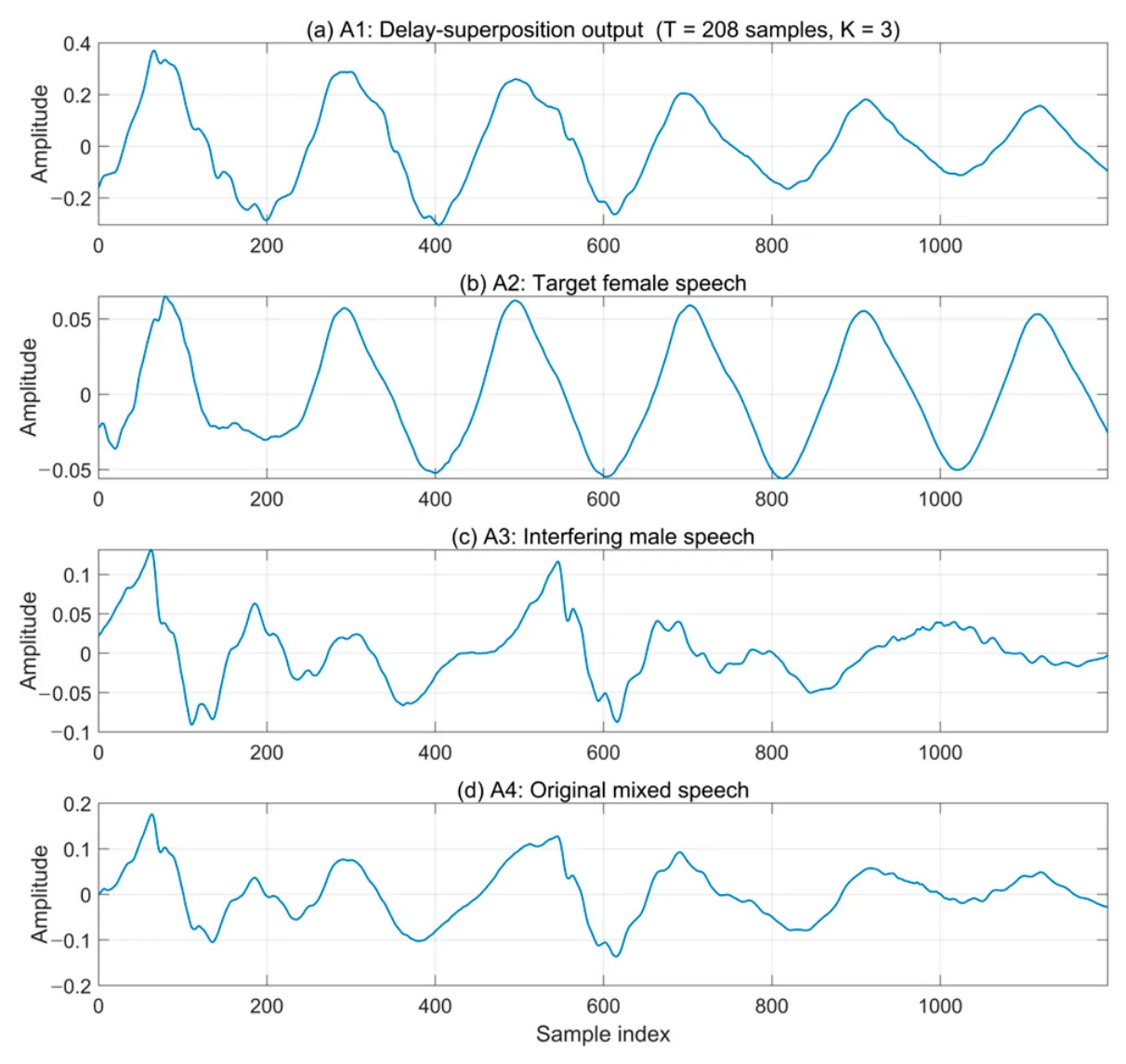
Illustration of the pitch-period-based delay-superposition process. A2 represents the target female speech component, A3 represents the interfering male speech component, and A4 represents their mixture. A1 is obtained by superimposing period-shifted copies of A4 using a target-related pitch period of *T* = 208 samples. Because the target component exhibits strong local quasi-periodicity at this delay, its periodic structure is constructively reinforced, whereas the mismatched interfering component is less consistently enhanced. The four signals are displayed separately for improved visualization.

For the vowel components, which exhibit strong quasi-periodicity, we implemented the DSA described in Section 2.2. Unlike the high-pass filtering effect of the differential method, DSA functions as a pitch-synchronous comb-like operation to constructively reinforce target-related periodic components. The delay-superposition operation follows Equations (3) and (4), where *x*[*n*] denotes the discrete mixed-speech signal, *n* is the sample index, *T* is the candidate delay in samples corresponding to a target-related pitch period, *k* is the superposition index, and *K* is the maximum number of superposition iterations. Figure 3 shows a simple example of the delay superposition method. Because the pitch period varies across speakers and over time, the algorithm did not rely on a single utterance-level delay. Instead, a set of candidate delay values was evaluated within a predefined search range. For each candidate *T*, delay-superposition processing was performed, and the resulting local segments were evaluated using the enhancement criterion described in Section 2.2.3. Segments exhibiting sufficient constructive enhancement were retained for subsequent reconstruction. Thus, pitch variability was accommodated through multi-delay candidate searching and local segment selection rather than through continuous frame-by-frame F0 tracking. For the male speakers, the delay lag *T* was searched within the range of 200–500 samples, corresponding to approximately 80–200 Hz at the sampling rate of 40 kHz, with *K* = 2~5 superposition iterations. For the female speakers, the delay range was 150–300 samples, corresponding to approximately 133–267 Hz at 40 kHz, with the same iteration strategy.

The main DSA does not employ an explicit frame-by-frame F0 estimator or a fixed analysis-frame length. Instead, *T* is treated directly as a candidate delay in samples and is evaluated within the predefined search range. Candidate delays are assessed according to the local enhancement criterion described in Section 2.2.3, and only segments satisfying the amplitude-enhancement criterion are retained. Therefore, no conventional frame-based overlap-add operation is used; the retained segments are incorporated into the original waveform through the zero-crossing-based selective replacement procedure described below.

**Figure 4:**
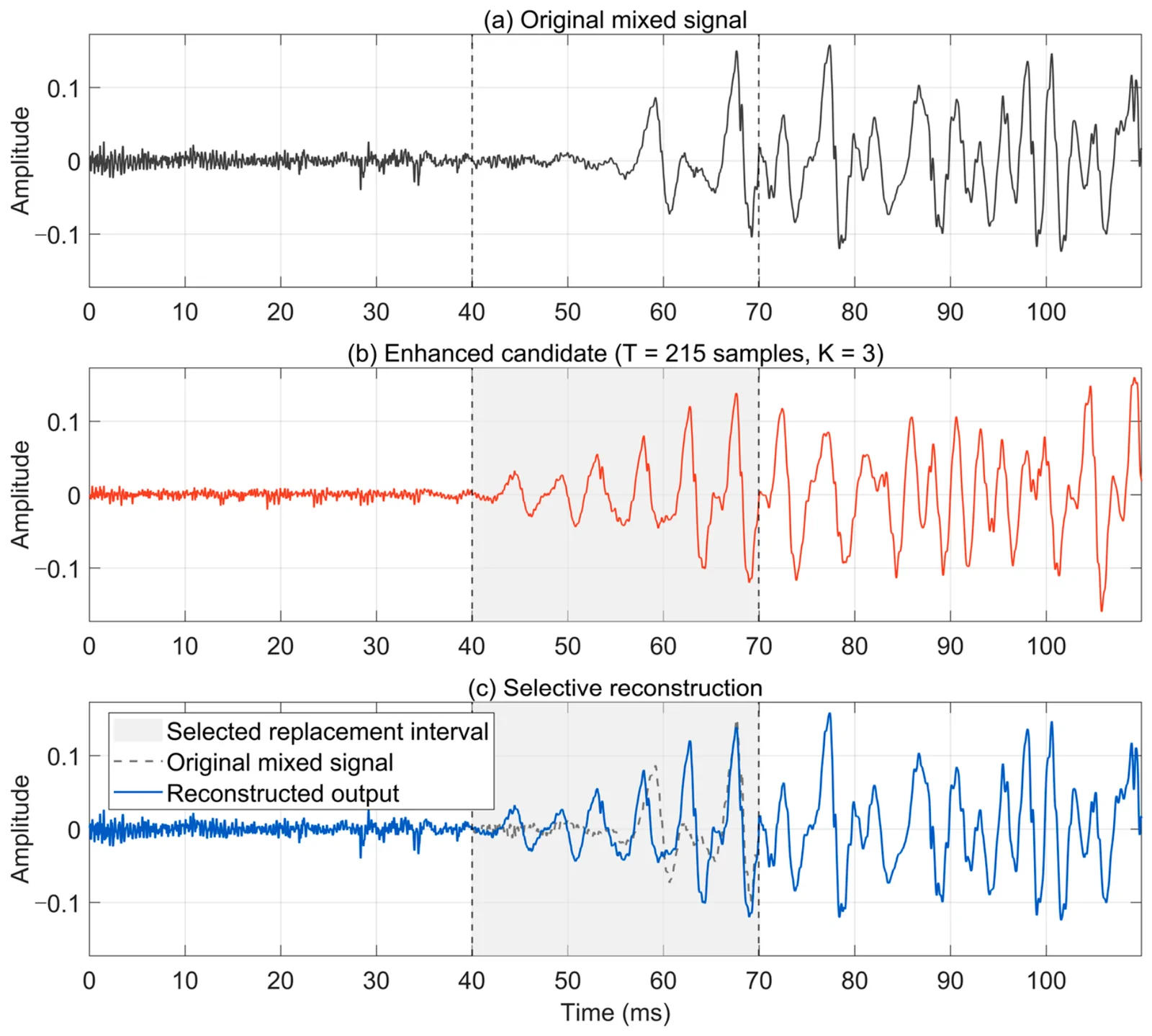
**Illustration of the selective waveform-reconstruction procedure.** (**a**) Original mixed speech signal. (**b**) Delay-superposition-enhanced candidate signal, with the shaded region indicating the segment selected for replacement. (**c**) Comparison between the original mixed signal and the reconstructed output. The original signal is retained outside the selected interval, whereas the corresponding segment within the selected interval is replaced by the enhanced candidate signal. Replacement boundaries are located near zero crossings to reduce waveform discontinuities.

To further optimize the enhanced vowel part, it is necessary to identify the amplitude maximum points of the vowel part at the corresponding positions of each audio signal to be processed for comparison, so as to retain the optimally enhanced part and replace the original mixed signal at that position. The comparison generally follows the following principles: the amplitude of the matched extreme points needs to be at least *N* times that of the extreme points of the original audio, and the maximum value among them is selected. Considering the problem of abrupt points that may occur during audio splicing, we only select the zero-crossing points where the amplitude is less than 0 for splicing to reduce errors. To a great extent, this avoids the noise caused by the abrupt points generated during splicing and improves the accuracy of the separated audio. We select the zero-crossing points before the extreme points from all the zero-crossing points, thereby ensuring that each extreme point has a corresponding matching zero-crossing point position. Select the starting and ending points of the processed audio and the corresponding zero-crossing points of the original audio for replacement (as shown in Figure 4).

#### 3.2.3. Spectrum Correlation-Based Quality Evaluation and Screening

To strictly evaluate the fidelity of the separated speech, we employed Spectral Correlation Analysis rather than simple time-domain waveform comparison. This approach focuses on the recovery of spectral structures (e.g., formant preservation) and is robust to minor phase shifts introduced during processing.

1. Data Alignment via Linear Interpolation: Since the extracted syllable segments often differ slightly in duration from the reference templates, we first applied linear interpolation to time-warp the extracted segment *x*_est_ to match the length of the ground-truth reference *x*_ref_. This ensures point-to-point correspondence in the subsequent spectral analysis.
2. Calculation of Spectral Similarity: We transformed both the aligned extracted signal *x_i_* and the original reference signal *x_j_* into the frequency domain using the Fast Fourier Transform (FFT). The spectral similarity coefficient $\rho_{ij}$ is defined as the Pearson correlation coefficient between their magnitude spectra $|X_{i}(f)|$ and $|X_{j}(f)|$:

(9) $$\rho_{ij}=\frac{\sum_{f}(|X_{i}(f)|-\mu_{i})\;\cdot \;(|X_{j}(f)|-\mu_{j})}{\sqrt{\sum_{f}(|X_{i}(f)|-\mu_{i}{)}^{2}}\;\cdot \;\sqrt{\sum_{f}(|X_{j}(f)|-\mu_{j}{)}^{2}}}$$

where |*X*(*f*)| represents the magnitude spectrum at frequency bin f, and μ denotes the mean value of the magnitude spectrum.

**Figure 5:**
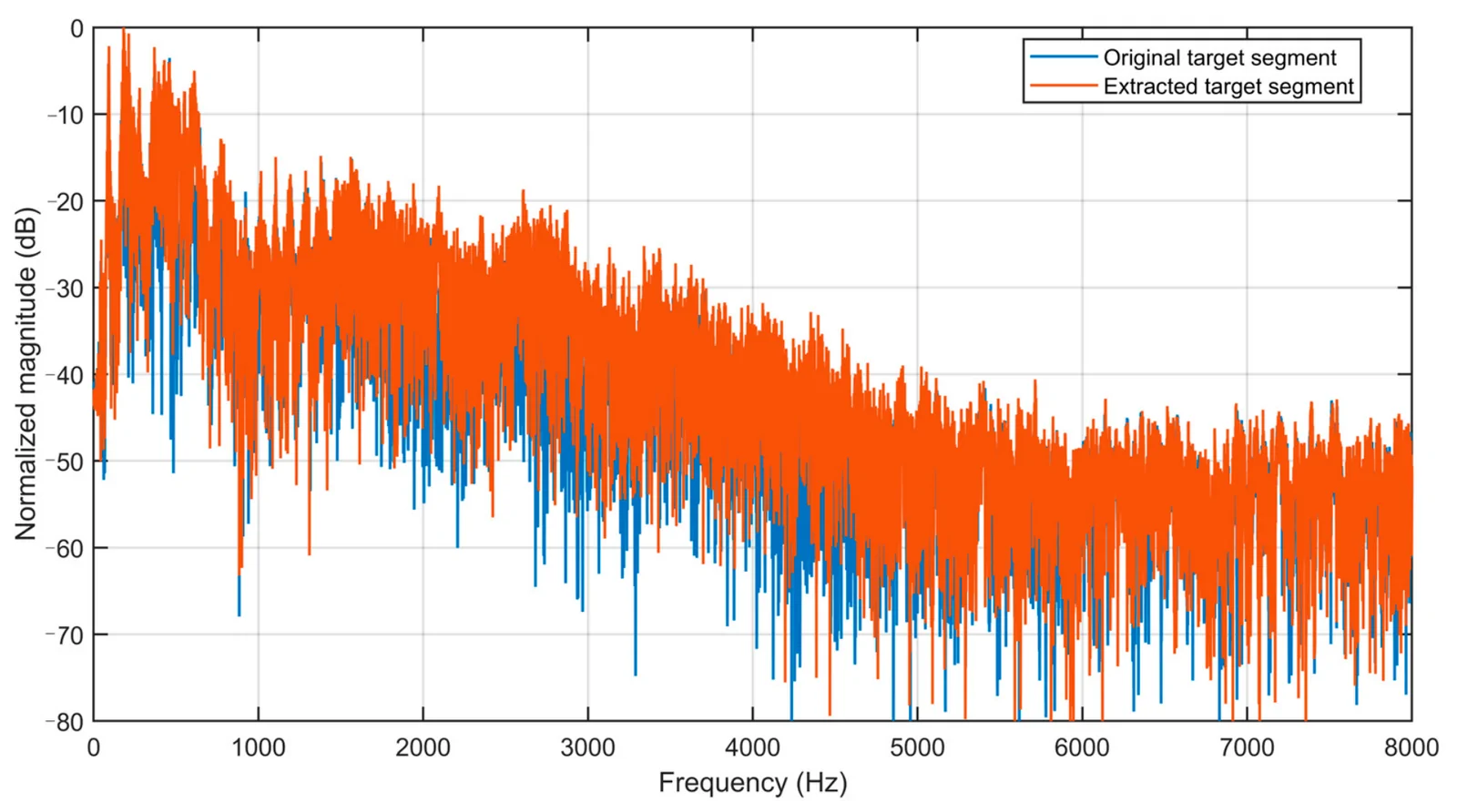
Spectral comparison of the monosyllabic segments of the sound segment with the sample mix.

For the complete dataset of *M* syllable segments, we constructed a correlation matrix $R\in R^{M\times M}$ to quantify the pairwise similarities:

(10) $$R\;=\;\left[\begin{array}{ccc} r_{11} & \ldots & r_{1M}\\ & \vdots & \\ r_{M1} & \ldots & r_{MM} \end{array}\right]$$

where the diagonal element *r_ii_* = 1 represents the autocorrelation of a fragment with itself. The off-diagonal element *r_ij_* indicates the normalized spectral similarity between the extracted fragment *i* and the reference template *j*. To ensure the high quality of the final dataset, we set a strict selection threshold. In the original spectral-similarity analysis, a threshold of ρ ≥ 0.80 was used to identify high-similarity segments for descriptive spectral comparison. Figure 5 presents an example of the spectral comparison between an original target segment and its corresponding extracted segment. This threshold was not used to exclude trials from the correlation-independent performance evaluation reported below. To complement this threshold-based analysis, an additional correlation-independent evaluation was conducted in which all processed trials, including unsuccessful cases, were retained without correlation-based exclusion.

### 3.3. Separation Results and Performance Analysis

Based on the above experimental procedure, this study employs the proposed method to conduct multi-turn speech separation experiments. The core objective was to determine whether the proposed delay-superposition strategy could selectively enhance a designated target speech signal in the presence of competing speech.

**Figure 6:**
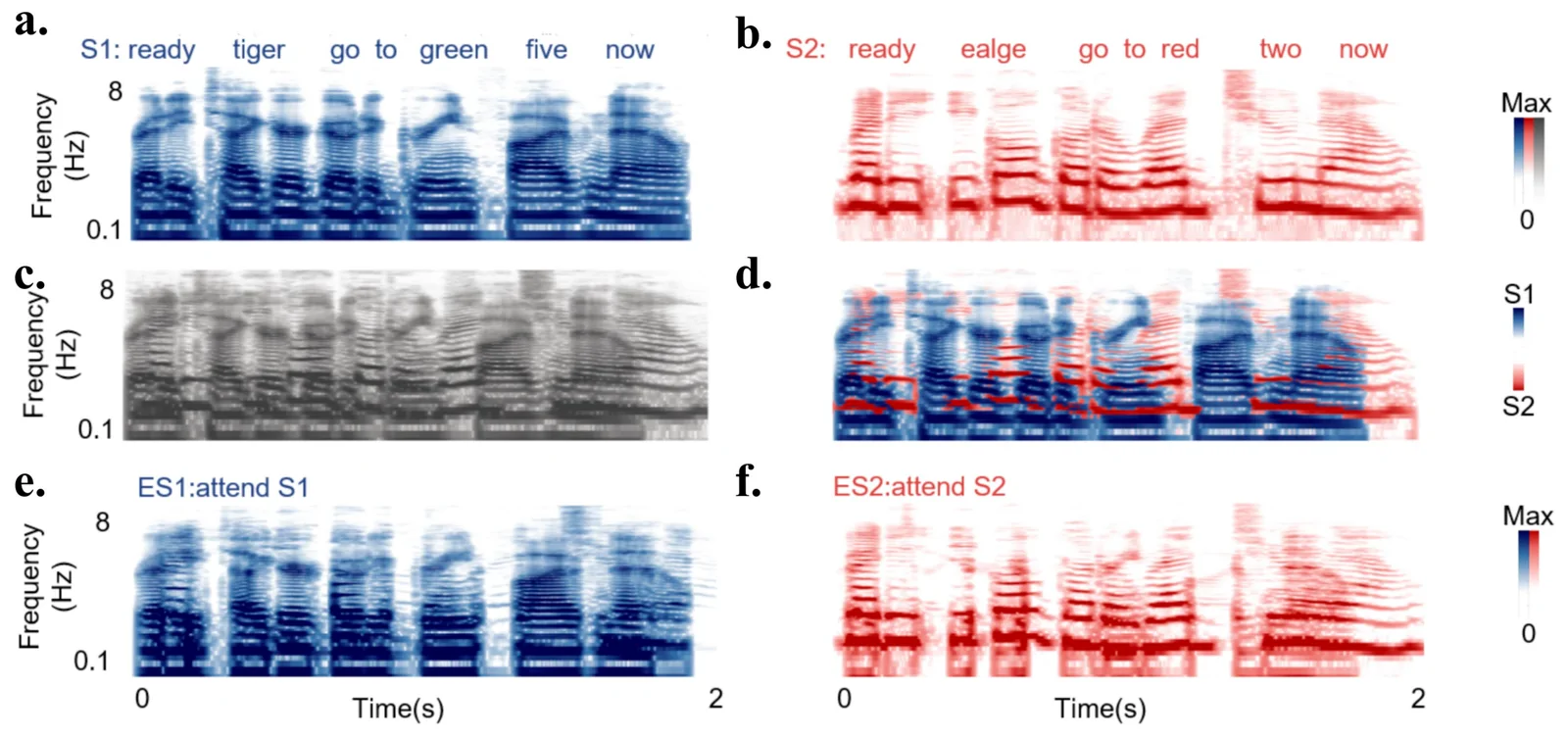
**Representative time-frequency comparison of clean, mixed, and enhanced speech signals.** (**a**,**b**) Spectrograms of the clean speech signals from speakers S1 and S2, respectively. (**c**,**d**) Spectrogram representations of the corresponding two-speaker mixed signal (MS). (**e**,**f**) Spectrograms of the enhanced outputs ES1 and ES2 obtained after delay-superposition processing, targeting S1 and S2, respectively.

We conducted 50 sets of comparative separation experiments to evaluate its performance. In the experiment, the delay superposition algorithm is adopted to achieve the separation and enhancement of the target speech signal in a multi-speaker environment. In this study, we conducted a total of 50 experiments. Figure 6 shows a typical set of experiments. For the representative experiment, speech samples from two speakers (S1 and S2) were selected from the standard CRM corpus [32]. Firstly, obtain the time-frequency diagrams of the clean speech of S1 and S2, respectively (Figure 6a,b), as well as the time-frequency diagrams of the mixed speech MS of the two (Figure 6c,d). The delay superposition algorithm adopts 3 superpositions. For the specific process of the superposition algorithm, please refer to the method. The extracted signals ES1 and ES2 of the two speakers (Figure 6e,f) are obtained, respectively. Through time-frequency domain comparative analysis, it is found that ES1 and S1 after delay enhancement processing exhibit significant similarity in the time-frequency domain. For quantitative analysis, the Normalized Cross-Correlation (NCC) is adopted for similarity evaluation. The NCC between the mixed voice MS and the pure voice S1 is 69.31%, while the NCC between the enhanced voice ES1 and the pure voice S1 increases to 90.42% (as compared in Figure 6a,e). Similarly, the NCC value of S2 before and after voice enhancement increases from 59.93% to 83.52% (as shown in the comparison between Figure 6b,f), with particularly significant improvements in temporal features. This result further demonstrates the effectiveness of the delay-superposition strategy for target-selective speech enhancement.

The representative results provide computational support for the biologically inspired design of the proposed delay-superposition strategy. The precedence (Haas) effect reflects the auditory system’s sensitivity to the temporal relationship between direct and delayed acoustic information, providing a functional motivation for the proposed framework [23,24,25]. In the present method, this principle is translated into a pitch-synchronous delay-superposition operation that reinforces periodic target components while relatively suppressing mismatched interference. Through this process, the formant-related structure of the target speaker’s voiced speech can be enhanced. This behavior shows a functional correspondence with auditory temporal integration and selective enhancement, although it should not be interpreted as a precise one-to-one physiological mapping.

**Figure 7:**
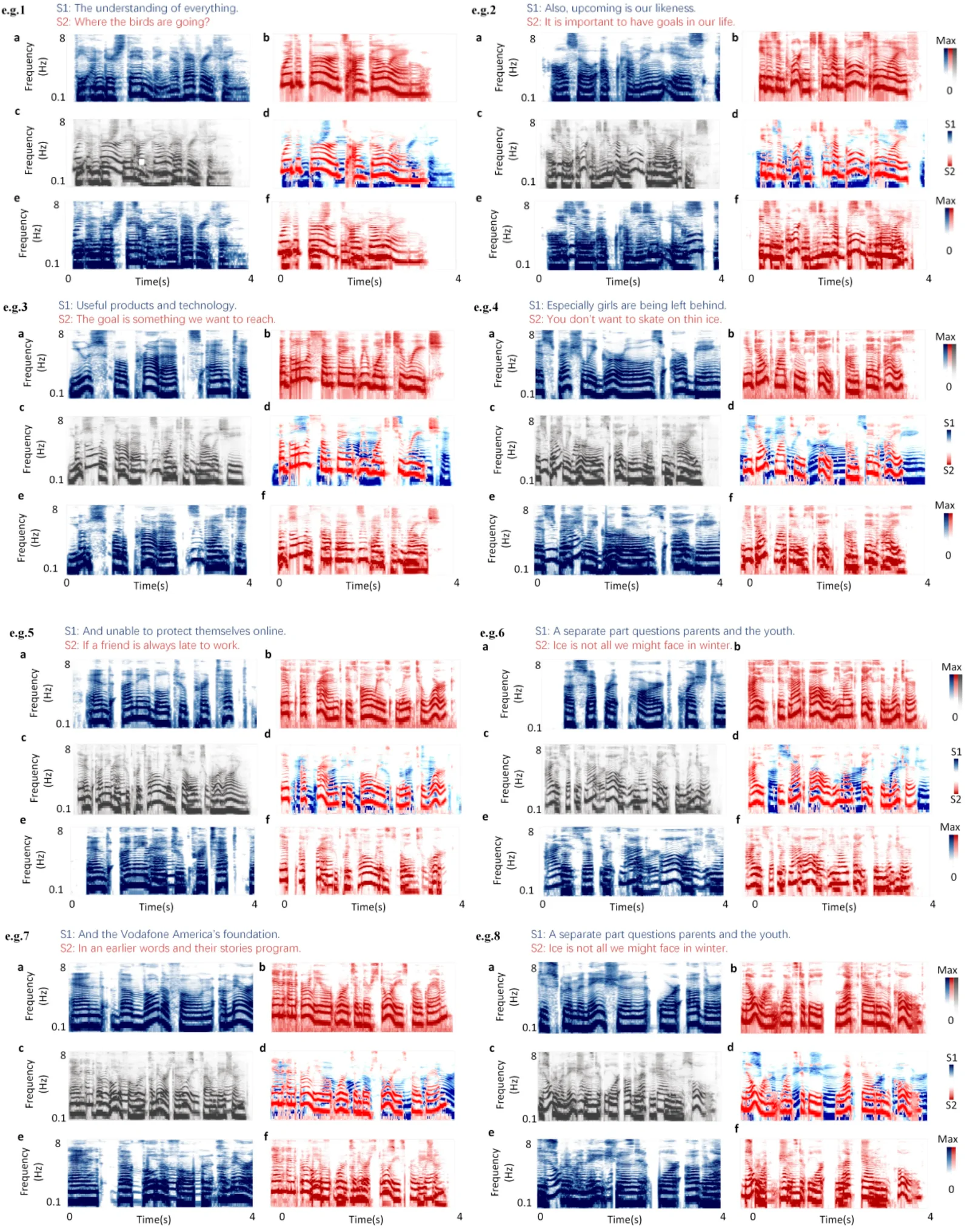

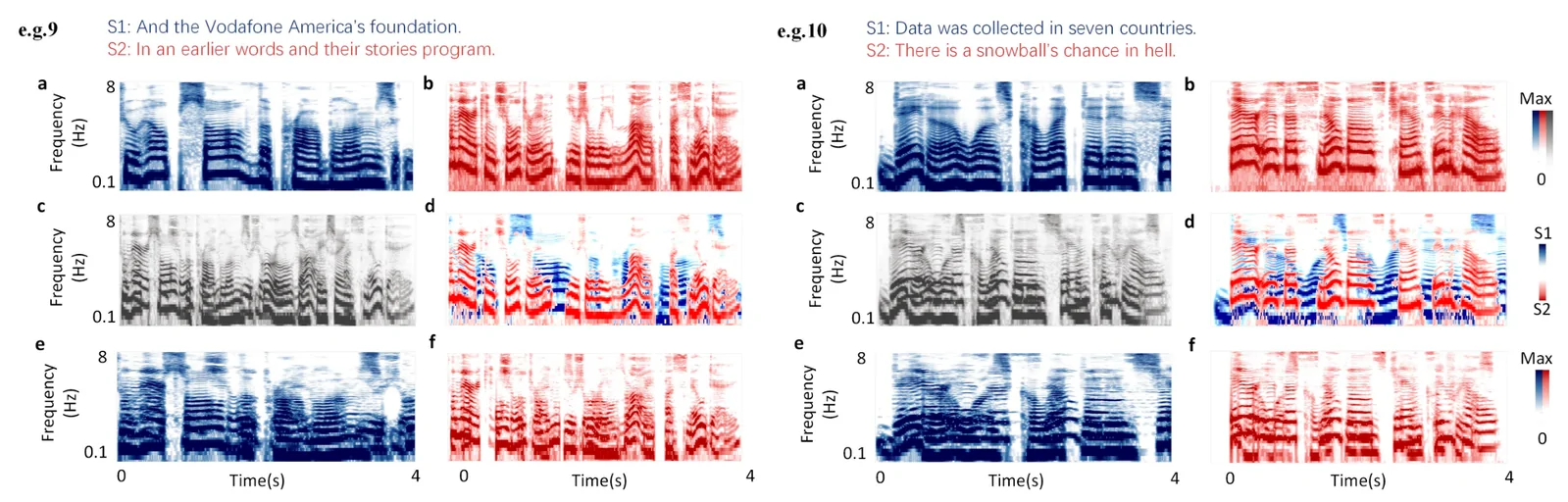
**Representative time-frequency results from ten two-speaker mixtures** (Examples 1–10, each representing a distinct case from the experimental set). For each example, panels (**a**) and (**b**) show the spectrograms of the two clean speech signals, panels (**c**) and (**d**) show the corresponding mixed-speech representation, and panels (**e**) and (**f**) show the enhanced outputs obtained after delay-superposition processing.

The successful separation observed in this representative case was further examined across additional speech mixtures. Several representative examples from the original experimental set were selected to qualitatively illustrate the algorithm’s behavior under varying fundamental-frequency characteristics, as shown in Figure 7. Across these examples, the delay-superposition process consistently enhanced target-related time-frequency structures while suppressing mismatched interference.

To further assess speaker-pair robustness beyond the initial male-female configuration, we evaluated the DSA on six additional speaker pairs spanning male-male, female-female, and male-female conditions. Three mixtures were evaluated for each pair, and both speakers were alternately treated as the target, yielding 36 target-specific trials. Selective enhancement was quantified by ΔSIR, defined as the difference between the target-component gain and interference-component gain after delay-superposition processing. Positive ΔSIR indicates that the target component was preferentially reinforced relative to the competing speaker. The results are summarized in Table 1.

Across all 36 trials, the DSA produced a mean ΔSIR of 1.67 ± 1.94 dB, with a median of 1.55 dB and an overall positive-enhancement success rate of 88.9%. Positive mean ΔSIR values were observed for all six speaker pairs. The male-male pair achieved a 100% success rate, while the four male–female pairs achieved success rates ranging from 66.7% to 100%. Greater variability was observed for the female-female pair, indicating that enhancement stability may decrease when competing speakers exhibit more similar periodic characteristics. This result also indicates an important operating boundary of the present method: selectivity becomes less stable when the target and interfering speakers exhibit strongly overlapping pitch-related periodicities. These additional results extend the evaluation beyond the original single male-female configuration and demonstrate that the pitch-related enhancement mechanism can operate across multiple speakers and speaker-pair conditions, while also revealing reduced selectivity for some acoustically similar speaker combinations. Importantly, all 36 target-specific trials were included in this analysis, including trials with negative ΔSIR values. No trial was excluded on the basis of spectral correlation or enhancement performance, thereby allowing both successful and unsuccessful cases to be represented in the reported statistics.

To further position the proposed method relative to established speech-processing approaches, we compared the DSA with three representative baselines using the same 36 target-specific trials described above. A conventional pitch-synchronous comb filter was selected as the most directly related periodicity-based baseline, an enrollment-based Wiener filter was included as a lightweight classical method, and a lightweight BiLSTM mask estimator was used as a neural baseline. All methods were evaluated using the same target/interference definitions and the same ΔSIR criterion, with all trials retained regardless of performance. Computational inference time was also recorded to provide an indication of processing complexity. The results are summarized in Table 2.

As shown in Table 2, the proposed DSA achieved the highest mean ΔSIR (1.67 ± 1.94 dB) and median ΔSIR (1.55 dB) among the four evaluated methods. The conventional pitch-synchronous comb filter provided a lower mean enhancement of 0.99 ± 1.05 dB. The Wiener-filter baseline produced a smaller mean ΔSIR of 0.36 ± 0.59 dB, while the lightweight BiLSTM baseline showed only limited target-selective improvement under the present training configuration. In terms of computational cost, the proposed DSA required substantially less inference time than the Wiener and neural baselines, although the simple comb filter remained the fastest method. These results suggest that the proposed strategy provides a favorable trade-off between target-selective enhancement, computational simplicity, and transparent signal-processing structure.

Finally, a controlled ablation analysis was performed on the same 36 target-specific trials to examine the respective contributions of the major processing components. Starting from the complete framework, the first-order differential pathway, delay-superposition pathway, and extreme-value screening stage were individually removed while the remaining settings were kept unchanged. ΔSIR was used to characterize target-selective enhancement, while STOI was used as an intelligibility-oriented measure. The results are summarized in Table 3.

The ablation results indicate that the individual components contribute differently to target selectivity and speech intelligibility. Removing the DSA reduced the mean ΔSIR from 1.67 dB to approximately 0 dB, indicating that the delay-superposition pathway is the principal contributor to target-selective enhancement. In contrast, removing the differential pathway reduced STOI from 0.528 to 0.473, suggesting that this pathway contributes to speech intelligibility. Removing the extreme-value screening stage produced a mean ΔSIR of 1.21 dB and STOI of 0.485, indicating that the screening operation acts primarily as a conservative local-selection constraint rather than directly maximizing global objective scores. Notably, the configuration without DSA retained a relatively high STOI of 0.518 but showed essentially no target-selective improvement, illustrating that intelligibility preservation and target-speaker selectivity represent distinct objectives in the present framework.

## 4. Biological Motivation and Perceptual Relevance

Having demonstrated the engineering efficacy of the proposed DSA in signal separation, we further examine its relationship with perceptual and biological characteristics of human auditory processing. This section investigates the perceptual relevance of the dual-pathway design through behavioral experiments and evaluates how the proposed computational framework corresponds to selected characteristics of auditory perception and selective attention.

First, through psychoacoustic substitution experiments, we examine the perceptual relevance of differentiated processing for vowels and consonants. Second, we use a selective-attention listening experiment to examine how behavioral performance changes with increasing competing-speech complexity. Finally, we compare these perceptual observations with the signal-processing behavior of the proposed framework at a functional level. These experiments are intended to assess the perceptual relevance of the engineering design rather than to establish neural equivalence or direct physiological validation.

### 4.1. Speech Perception Experiment

The dual-pathway design of the proposed model is motivated by the distinct temporal-acoustic characteristics of vowels and consonants. Voiced vowels generally exhibit sustained quasi-periodic structure, stable amplitude envelopes, and prominent formant patterns, providing important fundamental-frequency and prosodic information [33,34,35]. In contrast, many consonantal cues depend strongly on rapid acoustic transitions and millisecond-level transient features [36]. Based on these complementary characteristics, vowel- and consonant-related components are processed through separate pathways in the proposed framework. To evaluate the perceptual relevance of this differentiated processing strategy, we conducted an auditory experiment using acoustic substitution technology. Specifically, to quantify the perceptual impact of consonant variation on speech intelligibility, we implemented a controlled substitution protocol comprising three key stages:

(1). Data Preparation: Select multiple speakers (10 people) to record the same sentences containing a relatively large number of consonants (such as “The quick brown fox jumps over the lazy dog”). Extract the consonants (such as /*p*/, /*t*/, /*k*/, etc.) of each speaker from the following copy and build a consonant library.
(2). Stimulus generation: Selected consonants in the target speaker’s utterance were replaced by the corresponding consonants produced by another speaker, generating cross-speaker consonant-substitution stimuli.
(3). Clarity evaluation: Twenty listeners evaluated the perceived clarity of the generated speech samples on a five-point scale. All listeners had normal hearing, with pure-tone hearing thresholds ≤ 20 dB HL over the tested frequency range. The experiment was conducted individually in a quiet laboratory environment. Stimuli were presented binaurally through Sennheiser HD 280 Pro headphones at a fixed nominal presentation level of approximately 65 dB SPL. Clarity ratings were averaged across listeners and normalized to the maximum score according to $\mathrm{Clarity}(\%)\;=\;(\bar{x}/5)\;\times \;100\%$, where $\bar{x}$ denotes the mean clarity rating. The two substitution conditions were compared using a paired-samples *t*-test, with statistical significance defined as *p* < 0.05.

**Figure 8:**
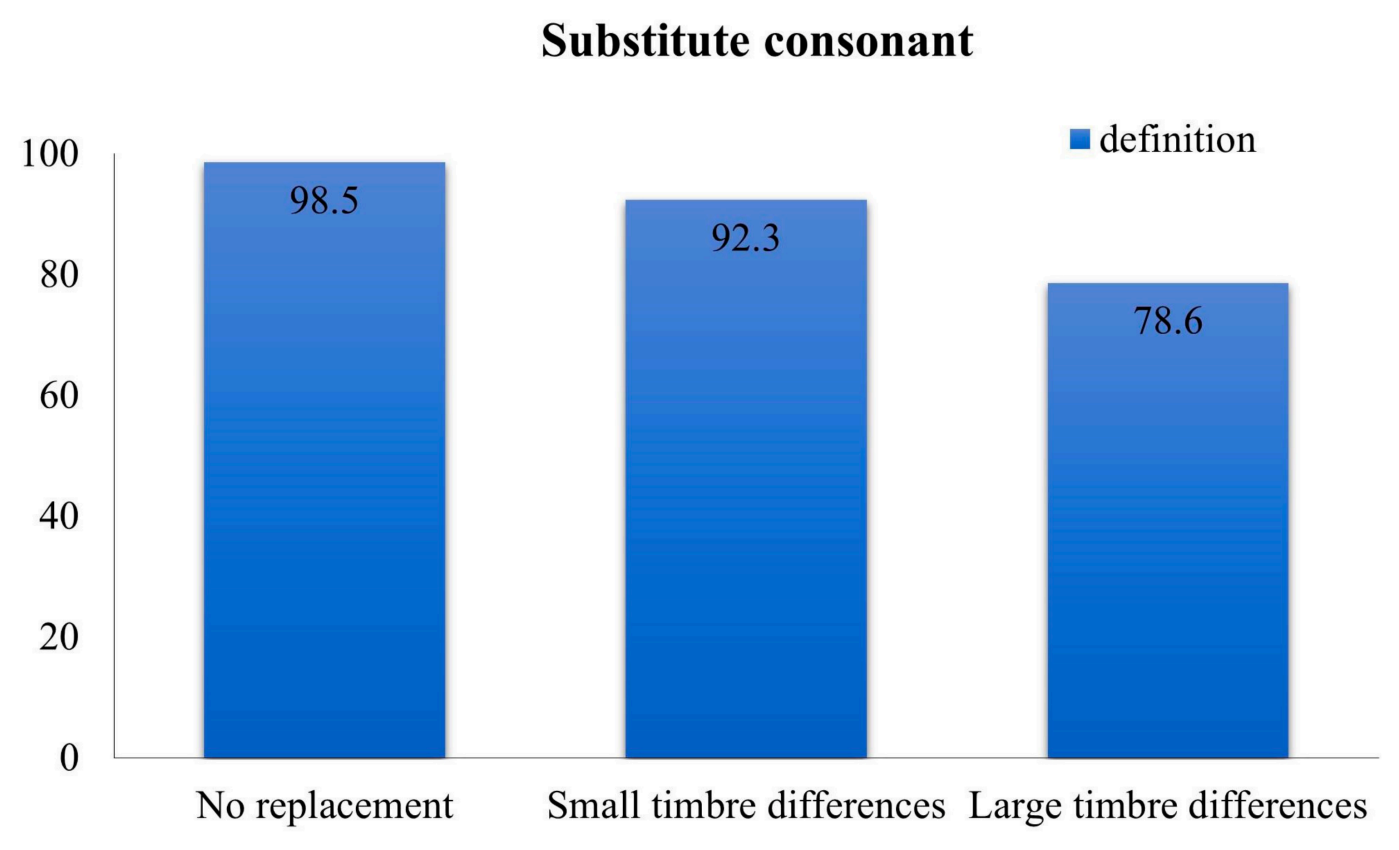
**Replacement consonant experiment.** If the replaced consonant is similar to the original consonant (for example, in the case of speakers of the same gender or with similar accents), the clarity may decrease slightly, with a speech clarity of 92.30%. If the replaced consonants come from speakers with significant phonetic differences (such as different genders or large accent differences), the clarity may decrease more significantly, namely to 78.60% of the speech intelligibility.

The experimental results shown in Figure 8 indicate that speech remained perceptually intelligible after cross-speaker consonant substitution, although the degree of intelligibility depended on the acoustic similarity between the original and replacement consonants. When consonants were replaced by acoustically similar tokens, such as those produced by speakers of the same gender or with similar accents, speech clarity remained relatively high at 92.30%. When the replacement consonants differed more substantially in speaker characteristics, speech intelligibility decreased to 78.60%. The difference in clarity between the two substitution conditions was statistically significant (paired-samples t-test, *p* = 0.012). These results support the perceptual relevance of treating transient consonant information separately from the more stable periodic structure of vowels. Accordingly, the experiment provides behavioral support for the differentiated processing strategy adopted in the proposed biomimetic framework, while not implying that the computational operations directly reproduce the underlying neural circuitry.

### 4.2. Selective-Attention Listening Experiment

Building on the bio-inspired hierarchical framework described above, which processes consonant and vowel information through complementary pathways, we further investigated whether a similar parallel-processing characteristic could be observed in human listening under selective-attention conditions resembling a real-world “cocktail party” scenario. To examine the cognitive and perceptual relevance of this processing strategy, we conducted a listening experiment in which participants attended to a designated target speaker while competing speech was simultaneously presented.

The experimental mixtures consisted of target speech produced by Speaker 1 and interfering speech produced by Speakers 2–5. Two distractor conditions were constructed. In the first condition, the target speech was a complete sentence, while the interfering sources consisted of monosyllabic neutral words, such as “hi,” “car,” and “do.” In the second condition, the same target speech was retained, whereas the interfering sources were replaced by highly salient words, such as participant names and warning-related expressions. Each condition contained 50 speech mixtures. The source levels were balanced within ±2 dB, and the interfering words were randomly embedded within 30–70% of the duration of the target utterance to provide variable temporal overlap between target and interfering speech.

The same 20 listeners who participated in Experiment 1 also completed this experiment. All participants satisfied the hearing criteria described in Section 4.1, and the same listening setup and presentation conditions were used. A double-blind procedure was adopted. Participants were instructed to attend to and repeat the target speech without prior information about the speaker characteristics, while also reporting interfering words that they could identify. Two behavioral measures were recorded: (1) interference-source recognition accuracy and (2) semantic-memory accuracy. For each participant, recognition accuracy and semantic-memory accuracy were calculated as the number of correct responses divided by the total number of trials and expressed as percentages.

**Figure 9:**
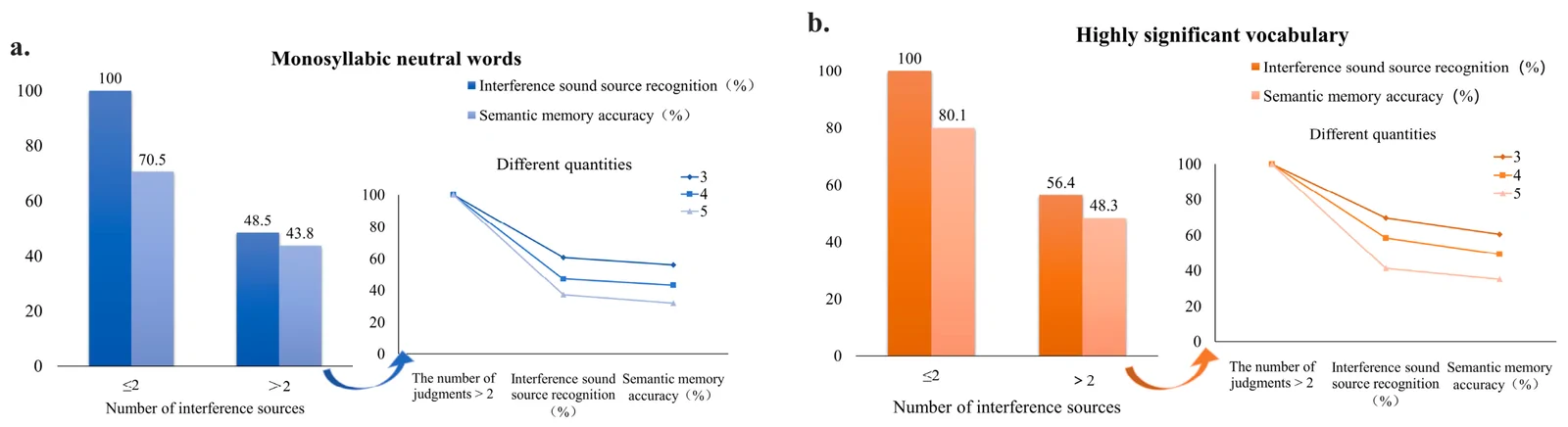
**Comparison between Selective Auditory Focus and Background Sound Recognition.** (**a**). Monosyllabic neutral word distractors: Near-perfect distractor recognition (100%) and 70.5% semantic memory accuracy for ≤2 distractor sources; recognition (48.5%) and memory (43.8%) decline sharply when sources > 2. (**b**). Highly salient word distractors: Near-perfect distractor recognition (100%) and higher semantic memory accuracy (80.1%) for ≤2 distractor sources; recognition (56.4%) and memory (48.3%) decline (less steeply than monosyllabic neutral words) when sources > 2.

As shown in Figure 9, when monosyllabic neutral words served as distractors, interference-source recognition accuracy reached 100% and semantic-memory accuracy was 70.50% when the number of distractor sources did not exceed two. When the number of distractor sources increased to 3–5, recognition accuracy decreased to 48.50%, while semantic-memory accuracy decreased to 43.80%. For highly salient distractors, interference-source recognition accuracy was 100% and semantic-memory accuracy was 80.10% when the number of distractors was ≤2, whereas these values decreased to 56.40% and 48.30%, respectively, when 3–5 distractor sources were present.

Overall, highly salient distractors produced higher recognition and semantic-memory performance than neutral monosyllabic distractors. As the number of interfering sources increased from ≤2 to 3–5, both source-recognition accuracy and semantic-memory accuracy decreased substantially. These results suggest that listeners can retain and process information from non-target speech while selectively attending to a target speaker, but this capacity decreases as the number of competing sources increases. The observed pattern supports a parallel auditory-processing strategy in which increasing acoustic competition places greater demands on attentional selection and prioritization of the target speech.

### 4.3. Functional Comparison with Auditory Selection

To further evaluate the biological plausibility of the proposed delay-superposition framework, we examined whether its selective enhancement behavior shows functional correspondence with human auditory selection. Previous studies have demonstrated that human listeners exhibit limited but robust target-speech recognition under competing-speech conditions, reflecting the constrained nature of auditory selective attention. Accordingly, we constructed a test set containing 50 male–female speech mixtures and alternately designated each speaker as the target signal. Following delay-superposition processing, the enhanced outputs were compared with the corresponding clean speech signals in the frequency domain.

**Figure 10:**
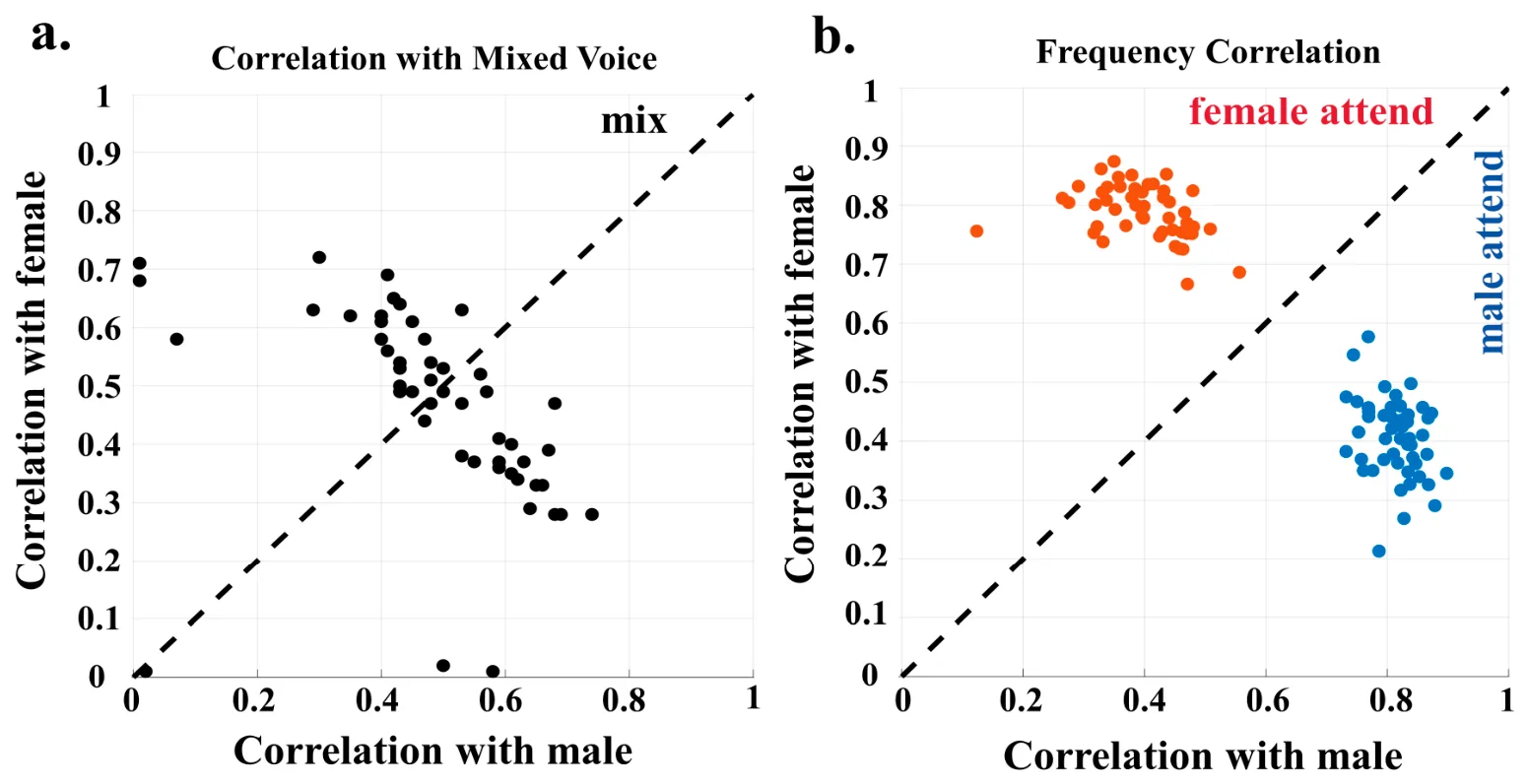
**Correlation-based visualization of target-speaker selectivity before and after delay-superposition processing.** (**a**). Scatter plot of time-domain correlation between speakers (S1, S2) and mixed signal (MS) across 50 experiments, showing low correlation in mixed speech. (**b**). Scatter plot of frequency-domain correlation between enhanced outputs (ES1, ES2) and original speakers (S1, S2), demonstrating effective speaker segregation: ES1 correlates highly with S1 and weakly with S2; ES2 correlates highly with S2 and weakly with S1.

As shown in Figure 10a, the original mixed speech exhibited comparable correlations with the two constituent speakers, indicating that the target component was not readily distinguishable in the mixture. After processing, the enhanced outputs exhibited stronger correlations with their designated target speakers, as shown in Figure 10b. Consistent with this qualitative observation, evaluation across all 36 target-specific trials yielded a mean ΔSIR of 1.67 ± 1.94 dB and a median ΔSIR of 1.55 dB, with positive target-selective enhancement observed in 88.9% of the trials.

These results demonstrate that the proposed algorithm produces a target-selective enhancement effect that is functionally analogous to an important characteristic of human selective hearing. The comparison should not be interpreted as a numerical equivalence between spectral correlation and human recognition accuracy, because the two measurements quantify different phenomena. Instead, the results support the broader biomimetic interpretation that biologically inspired temporal integration can be abstracted into a computational mechanism for selectively strengthening a target acoustic representation.

## 5. Discussion

In this study, we developed an interpretable biomimetic auditory framework by translating selected temporal-processing characteristics of human hearing into explicit signal-processing operations. The proposed delay-superposition strategy exploits the quasi-periodicity of vowel signals to produce constructive reinforcement of target components whose periods match the selected delay, whereas mismatched components are less coherently accumulated. In parallel, differential processing emphasizes rapidly varying transient features associated with consonants. The combination of these complementary pathways provides a computational abstraction of differentiated auditory processing rather than a direct reproduction of neural circuitry.

The experimental results demonstrate that this biologically inspired strategy can produce substantial target-speech enhancement in controlled two-speaker mixtures. In representative experiments, normalized cross-correlation with the clean target increased from 0.69 to 0.90 for one speaker and from 0.60 to 0.84 for the other. These findings indicate that temporal periodicity can serve as an effective biologically motivated cue for selectively enhancing target speech.

The psychoacoustic experiments further provide behavioral support for the differentiated processing strategy employed in the model. The consonant-substitution experiment indicates that transient consonant information can vary substantially while speech remains perceptually intelligible, whereas the selective-attention experiment demonstrates that auditory performance deteriorates as the number of competing sources increases. Together, these observations are consistent with the general idea that human auditory perception combines parallel acoustic processing with selective allocation of limited attentional resources.

Importantly, the biological interpretation of the present model should be considered at the functional and computational levels. The delay-superposition algorithm is inspired by temporal integration phenomena associated with the Haas effect, but the present experiments do not establish that the human auditory system performs the same mathematical operation. Accordingly, the primary contribution of this study is the translation of an auditory biological principle into an interpretable engineering architecture. This distinction is important for biomimetic modeling because it allows biological inspiration to guide system design without requiring a one-to-one correspondence between an engineering algorithm and its underlying neural implementation.

The present study also has several limitations. First, the main separation experiments were conducted in controlled two-speaker mixtures, and further evaluation is required in environments containing more speakers, reverberation, and non-speech noise. Second, the current delay-selection strategy relies on predefined target-related candidate ranges rather than continuous adaptive F0 tracking. Future work will focus on estimating and tracking time-varying target pitch directly from the mixture or from more general target-speaker cues. Third, the biological plausibility of the framework is currently supported primarily at computational and behavioral levels rather than through direct neurophysiological measurements. Fourth, the current waveform-reconstruction strategy relies on negative-going zero-crossing points without overlap-add or window-based cross-fading. Therefore, residual click-like artifacts or local phase discontinuities may still occur in non-stationary speech, and more advanced boundary-smoothing strategies will be considered in future work. Future studies should therefore evaluate the proposed mechanism using more diverse datasets, standardized speech-separation and intelligibility metrics, and, where possible, direct comparisons with experimentally measured auditory neural responses.

## 6. Conclusions

This study proposed an interpretable biomimetic auditory-processing framework for target-speech enhancement in competing-speech environments. The framework translates two complementary characteristics of auditory processing into explicit computational operations: differential processing is used to emphasize rapidly varying consonant features, whereas a delay-superposition mechanism inspired by the Haas effect exploits the quasi-periodicity of voiced speech to reinforce target vowel structures.

Across all 36 target-specific evaluation trials, the proposed method achieved a mean ΔSIR of 1.67 ± 1.94 dB and a median ΔSIR of 1.55 dB, with positive target-selective enhancement observed in 88.9% of the trials. These results demonstrate that the proposed pitch-synchronous delay-superposition mechanism can selectively reinforce the designated target speech across different speaker-pair conditions. Psychoacoustic experiments further supported the functional relevance of differentiated speech-component processing and selective auditory attention.

Rather than claiming to reproduce the complete neural mechanism of the cocktail party effect, the proposed approach demonstrates how biological auditory principles can be abstracted into a transparent and testable engineering framework. The results therefore provide a biomimetic pathway toward interpretable auditory sensing and speech-processing systems. Future work will focus on adaptive parameter estimation, more complex multi-speaker and noisy environments, standardized intelligibility evaluation, and closer comparison with neurophysiological measurements.

## Figures and Tables

**Table 1:** Additional multi-speaker evaluation of DSA selectivity across same- and cross-gender CRM speaker pairs.

Pair Type	Trials	ΔSIR (dB), Mean ± SD	Median ΔSIR (dB)	SI-SDRi, Mean ± SD (dB)	Success Rate (%)
Male–male	6	2.07 ± 1.22	2.02	6.40 ± 2.26	100.0
Female–female	6	2.39 ± 3.90	2.23	6.37 ± 3.19	83.3
Male–female	6	2.07 ± 1.60	2.11	5.34 ± 2.25	100.0
Male–female	6	0.77 ± 1.25	0.56	6.35 ± 1.92	66.7
Male–female	6	1.18 ± 0.70	1.00	4.47 ± 1.15	100.0
Male–female	6	1.51 ± 1.63	1.26	6.86 ± 1.36	83.3
Overall	36	1.67 ± 1.94	1.55	/	88.9%

Note: The "/" in the table indicates that the corresponding Pair Type is not applicable to the current metric.

**Table 2:** Comparison of the proposed DSA with representative classical and neural baseline methods on the 36-trial evaluation set.

Method	Type	ΔSIR, Mean ± SD (dB)	Median ΔSIR (dB)	Success Rate (%)	Mean Inference Time (ms)	Training Required
Conventional pitch-synchronous comb filter	Classical	0.99 ± 1.05	0.94	83.3	0.23	No
Enrollment-based Wiener filter	Classical	0.36 ± 0.59	0.41	75.0	6.05	No
Lightweight BiLSTM mask estimator	Neural	0.62 ± 0.57	0.64	55.6	37.30	Yes
Proposed DSA	Bio-inspired	1.67 ± 1.94	1.55	88.9%	0.67	No

**Table 3:** Ablation analysis of the main components of the proposed framework on the 36-trial evaluation set.

Configuration	ΔSIR, Mean ± SD (dB)	STOI, Mean ± SD
Full framework	1.67 ± 1.94	0.528 ± 0.088
*w*/*o* differential pathway	1.20 ± 2.01	0.473 ± 0.091
*w*/*o* DSA	0.00 ± 0.36	0.518 ± 0.093
*w*/*o* extreme-value screening	1.21 ± 1.75	0.485 ± 0.099

## Data Availability

The raw data supporting the conclusions of this article will be made available by the authors on request.
